# Early postnatal soluble FGFR3 therapy prevents the atypical development of obesity in achondroplasia

**DOI:** 10.1371/journal.pone.0195876

**Published:** 2018-04-13

**Authors:** Celine Saint-Laurent, Stephanie Garcia, Vincent Sarrazy, Karine Dumas, Florence Authier, Sophie Sore, Albert Tran, Philippe Gual, Isabelle Gennero, Jean-Pierre Salles, Elvire Gouze

**Affiliations:** 1 Université Côte d’Azur, CNRS, Inserm, iBV, Nice, France; 2 Université Côte d’Azur, CHU, Inserm, C3M, Nice, France; 3 University of Paul Sabatier Toulouse III, Inserm, CPTP, Toulouse, France; 4 Biochemistry Laboratory, Institut Federatif de Biologie, Toulouse University hospital, Toulouse, France; 5 Endocrine, Bone Diseases, and Genetics Unit, Children's Hospital, Toulouse University Hospital, Toulouse, France; Max Delbruck Centrum fur Molekulare Medizin Berlin Buch, GERMANY

## Abstract

**Background:**

Achondroplasia is a rare genetic disease is characterized by abnormal bone development and early obesity. While the bone aspect of the disease has been thoroughly studied, early obesity affecting approximately 50% of them during childhood has been somewhat neglected. It nevertheless represents a major health problem in these patients, and is associated to life-threatening complications including increasing risk of cardiovascular pathologies. We have thus decided to study obesity in patients and to use the mouse model to evaluate if soluble FGFR3 therapy, an innovative treatment approach for achondroplasia, could also impact the development of this significant complication.

**Methods and findings:**

To achieve this, we have first fully characterized the metabolic deregulations in these patients by conducting a longitudinal retrospective study, in children with achondroplasia Anthropometric, densitometric measures as well as several blood parameters were recorded and compared between three age groups ranging from [0–3], [4–8] and [9–18] years old. Our results show unexpected results with the development of an atypical obesity with preferential fat deposition in the abdomen that is remarkably not associated with classical complications of obesity such as diabetes or hypercholosterolemia. Because it is not associated with diabetes, the atypical obesity has not been studied in the past even though it is recognized as a real problem in these patients. These results were validated in a murine model of achondroplasia (*Fgfr3*^*ach/+*^) where similar visceral adiposity was observed. Unexpected alterations in glucose metabolism were highlighted during high-fat diet. Glucose, insulin or lipid levels remained low, without the development of diabetes. Very interestingly, in achondroplasia mice treated with soluble FGFR3 during the growth period (from D3 to D22), the development of these metabolic deregulations was prevented in adult animals (between 4 and 14 weeks of age). The lean-over-fat tissues ratio was restored and glucose metabolism showed normal levels. Treating *Fgfr3*^*ach/+*^ mice with soluble FGFR3 during the growth period, prevented the development of these metabolic deregulations in adult animals and restored lean-over-fat tissues ratio as well as glucose metabolism in adult animals.

**Conclusion:**

This study demonstrate that achondroplasia patients develop an atypical obesity with preferential abdominal obesity not associated with classical complications. These results suggest that achondroplasia induces an uncommon metabolism of energy, directly linked to the FGFR3 mutation. These data strongly suggest that this common complication of achondroplasia should be included in the clinical management of patients. In this context, sFGFR3 proved to be a promising treatment for achondroplasia by normalizing the biology at different levels, not only restoring bone growth but also preventing the atypical visceral obesity and some metabolic deregulations.

## Introduction

Achondroplasia, the most common form of short limb dwarfism, is a rare genetic disease for which there is no cure [1]. In most patients, a G380R substitution in the transmembrane domain of the fibroblast growth factor receptor 3 (FGFR3) (Fgfr3ach) results in a gain-of-function, prolonging the intracellular MAPK signaling [2, 3]. Unlike in other cell types, in the growth plate, the MAPK signaling is inhibitory and its subsequent constitutive activation results in the inhibition of chondrocyte proliferation and differentiation [4, 5]. Cells expressing the mutant receptor do not mature and are not replaced by mineralized bone matrix, impairing lengthening of the bones. We have recently successfully developed a recombinant protein treatment, using a decoy soluble FGFR3 (sFGFR3), restoring full bone growth in mice carrying the achondroplasia mutation (*Fgfr3*^*ach/+*^) [6] opening the way of potential treatment in humans.

However, when designing new innovative treatment approach, it is necessary to consider all aspects of the disease. Indeed, achondroplasia is also characterized by early obesity which represents a major health problem in these patients, affecting approximately 50% of them during childhood [7]. Obesity augments the morbidity associated with lumbar lordosis as well as the severity of orthopedic complications, increasing for example bearing weight on already fragile knees. It can also increase the risk of serious complications such as cardiovascular risks, obstructive sleep apnea or restrictive lung disease [8, 9]. The causes of this increased susceptibility to obesity in achondroplasia patients are not known [10]; it has been established for instance that they do not suffer from hormonal or neurological dysfunction that can lead to appetite deregulation such as hyperphagia [7]

Obese achondroplasia patients seem to suffer from associated metabolic complications. Although there are only three case reports in the literature, they all appear to describe the same type of metabolic complications with apparent dyslipidemia and low insulin levels [11–13]. One study from 1972 also suggests a tendency to glucose intolerance [11]. None of these reports however give indications on whether these complications are isolated and related to exogenous factors such as excessive caloric intake and/or decreased physical activity, or if they indeed reflect underlying defect in achondroplasia.

While bone growth alterations are well described and studied, these body weight issues and metabolic disturbances are not yet understood. In this context, understanding whether and how obesity and metabolic complications are also consequences of the FGFR3 mutation is an important question to solve before proposing a treatment. In this paper, we thus first characterized metabolic disturbances in children with achondroplasia leading to the preferential development of atypical abdominal obesity that is unexpectedly not associated with the concurrent development of diabetes or hypercholesterolemia. In *Fgfr3*^*ach/+*^ mice, alterations in glucose metabolism lead to similar visceral obesity development. The Fgfr3ach mutation also modifies the differentiation potential of mesenchymal stem cells in *Fgfr3*^*ach/+*^ mice. In mice treated with sFGFR3 during the growth period, for which bone growth is restored, these metabolic deregulations are prevented and the stem cell differentiation potentials are fully restored. sFGFR3 proves to be a promising treatment for achondroplasia not only restoring bone growth but also preventing the metabolic deregulations and the development of this unusual visceral obesity.

## Results

### Achondroplasia patients develop an excess of abdominal adipose tissue without classical complications

Subjects were children and adolescents with achondroplasia. They were included in a longitudinal, retrospective study conducted by the same observer for an average of 8.6 ± 5.6 years. Anthropometric measures and body composition were recorded from birth on during follow up visits and compared between three age groups ranging from [0–3], [4–8] and [9–18] years old. Several metabolic blood parameters were measured and compared in the different age groups. Blood values for visits under age of 3 were not considered because of the difficulty to restrict food intake in infants and thus control metabolic parameters at this young age.

**Fig 1:**
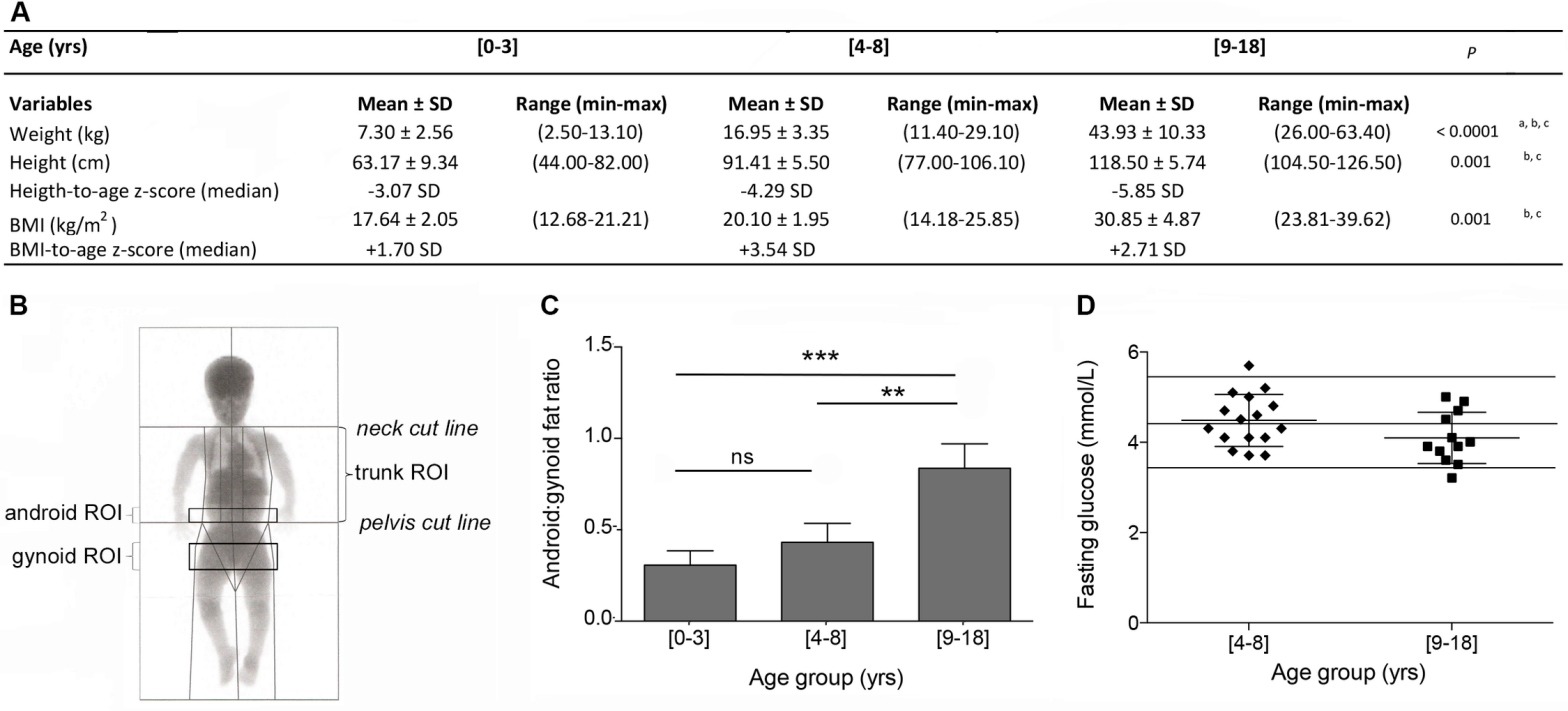
Children with achondroplasia preferentially develop abdominal obesity without an increase in blood glucose levels. (**A**), Weight, height and BMI measurements and corresponding height-to-age and BMI-to-age z-scores in the three age groups ranging (n = 73 data points in the [0–3] years old group, n = 61 data points in the [4–8] years old group and n = 36 data points in the [9–18] years old group). (**B**), Schematic representation of the different regions of interest (ROI) evaluated by DXA. (**C**), Android:gynoid fat ratio measurement in the three age groups ranging (n = 4 data points in the [0–3] years old group, n = 6 data points in the [4–8] years old group and n = 9 data points in the [9–18] years old group). (**D**), Plasmatic fasting glucose concentration in the two age groups ranging [4–8] and [9–18] years old (n = 16 data points in the [4–8] years old group and n = 12 data points in the [9–18] years old group). Horizontal lines represent normal values. Data are represented as mean ± SD, **p<0.01, ***p<0.001. Results of post hoc analyses: ^a^ significantly different between [0–3] and [4–8] groups; ^b^ significantly different between [0–3] and [9–18] groups; ^c^ significantly different between [4–8] and [9–18] groups.

It is known that achondroplasia patients not only display impaired growth but that they also have a tendency to gain excessive weight leading to overweight or even obesity [14]. As seen in Fig 1A, the BMI of patients with achondroplasia significantly increased during childhood, to reach a value of about 30 kg/m^2^ in the [9–18] years old group (P = 0.2407 between [0–3] and [4–8] groups, P<0.0001 comparing the [9–18] group to both other groups, Tukey's multiple comparisons test). A negative correlation was observed between BMI and height (Pearson r coefficient = -0.5660, P = 0.0021), with a tendency for the smallest children to have the highest BMI. No statistical differences were observed between males and females (S1 Table). To study their metabolic status and its evolution, densitometry analyses were first performed to determine body fat and lean mass distribution in the different age groups (Fig 1B). We observed that up to 8 years old, the total fat:lean ratio was relatively constant and that it rose significantly during adolescence, from 0.21 ± 0.02 and 0.20 ± 0.05 in the [0–3] and [4–8] age groups to 0.84 ± 0.29 in the [9–18] age group, respectively (P = 0.9954 between [0–3] and [4–8] groups, P = 0.0053 and P<0.0001 comparing [9–18] to [0–3] and [4–8] age groups, respectively, Tukey's multiple comparisons test) (S2 Table). Interestingly, as seen in Fig 1C, the increase in fat mass was not homogeneous and patients preferentially developed abdominal (android) fat mass (+204%) over hip (gynoid) fat mass (+55%) (P = 0.0974 between [0–3] and [4–8] groups, P = 0.0002 and P = 0.001 comparing [9–18] to [0–3] and [4–8] age groups, respectively, Tukey's multiple comparisons test). Concurrently, both android and gynoid lean masses did not vary during this period (S2 Table). Consequently, the fat:lean ratio significantly increased in the abdominal area throughout childhood (P = 0.3187 between [0–3] and [4–8] groups, P = 0.0924 and P<0.0001 comparing [9–18] to [0–3] and [4–8] age groups, respectively, Tukey's multiple comparisons test). The trunk, legs and arms followed a very similar trend with an increase in percent fat mass and decrease in percent lean mass from infancy to adulthood (S2 Table). Spinal bone mineral density (BMD) was determined between L1 and L4 after age of 3. In both age groups, BMD was found to be below age-appropriate normal range value[15] (0.511 ± 0.065 g/cm^2^ and 0.898 ± 0.223 g/cm^2^ in the [4–8] and [9–18] age groups, respectively, compared to 0.645 ± 0.071 g/cm^2^ and 0.913 ± 0.199 g/cm^2^ in the same age referenced groups).

Different blood parameters were compared between the [4–8] and [9–18] age groups. Unexpectedly, in both age groups and independently of their BMI, achondroplasia children displayed a tendency to low plasmatic total cholesterol (3.38 ± 0.36 mmol/L and 3.73 ± 0.44 mmol/L in the [4–8] and [9–18] age groups, respectively, with normal values being comprised between 3.90 and 5.70 mmol/L in children), and low triglycerides (0.56 ± 0.14 mmol/L and 0.63 ± 0.13 mmol/L in the [4–8] and [9–18] age groups, respectively, with normal values being comprised between 0.60 and 1.70 mmol/L in children). Similarly, fasting blood glucose (Fig 1D) and insulin levels were not increasing with age and remained within normal range (7.3 ± 5.4 mUI/L and 13.4 ± 3.4 mUI/L in the [4–8] and [9–18] age groups, respectively, with normal values being comprised between 2.6 and 16 mUI/L in children).

These results were confirmed by glucose levels obtained during oral glucose tolerance test (OGTT), that showed normal glucose levels at 0, 30 and 120 minutes following oral administration (4.53 ± 0.22 mmol/l at 0 min, 7.97 ± 1.72 mmol/l at 30 min and 5.17 mmol/l at 120 min). No statistical differences were found between both age groups. Due to high levels of hemolysis during the OGTT, no data were unfortunately available regarding insulin levels. All other blood parameters were within normal range (S3 Table).

### Metabolic alterations are observed in lean and obese *Fgfr3*^*ach/*+^ mice and are corrected by sFGFR3 treatment

To determine the role of FGFR3ach in this preferential development of visceral obesity in achondroplasia, transgenic *Fgfr3*^*ach/+*^ mice carrying the G380R mutation or their wild-type (WT) littermates were treated with sFGFR3 or vehicle for 3 weeks starting at day 3. Mice were then challenged with normal (ND) or high fat diet (HFD) starting at 4 weeks of age for a duration of 10 weeks to evaluate the development of obesity.

After weaning, at 4 weeks of age, as expected, untreated *Fgfr3*^*ach*/+^ mice had a 20.4% decrease in body weight compared to their WT littermates. This was associated with reduced lean and fat tissues (50% and 33.9% respectively). Treated animals displayed a 14.1% decrease in body weight compared to WT mice (P<0.0001).

**Fig 2:**
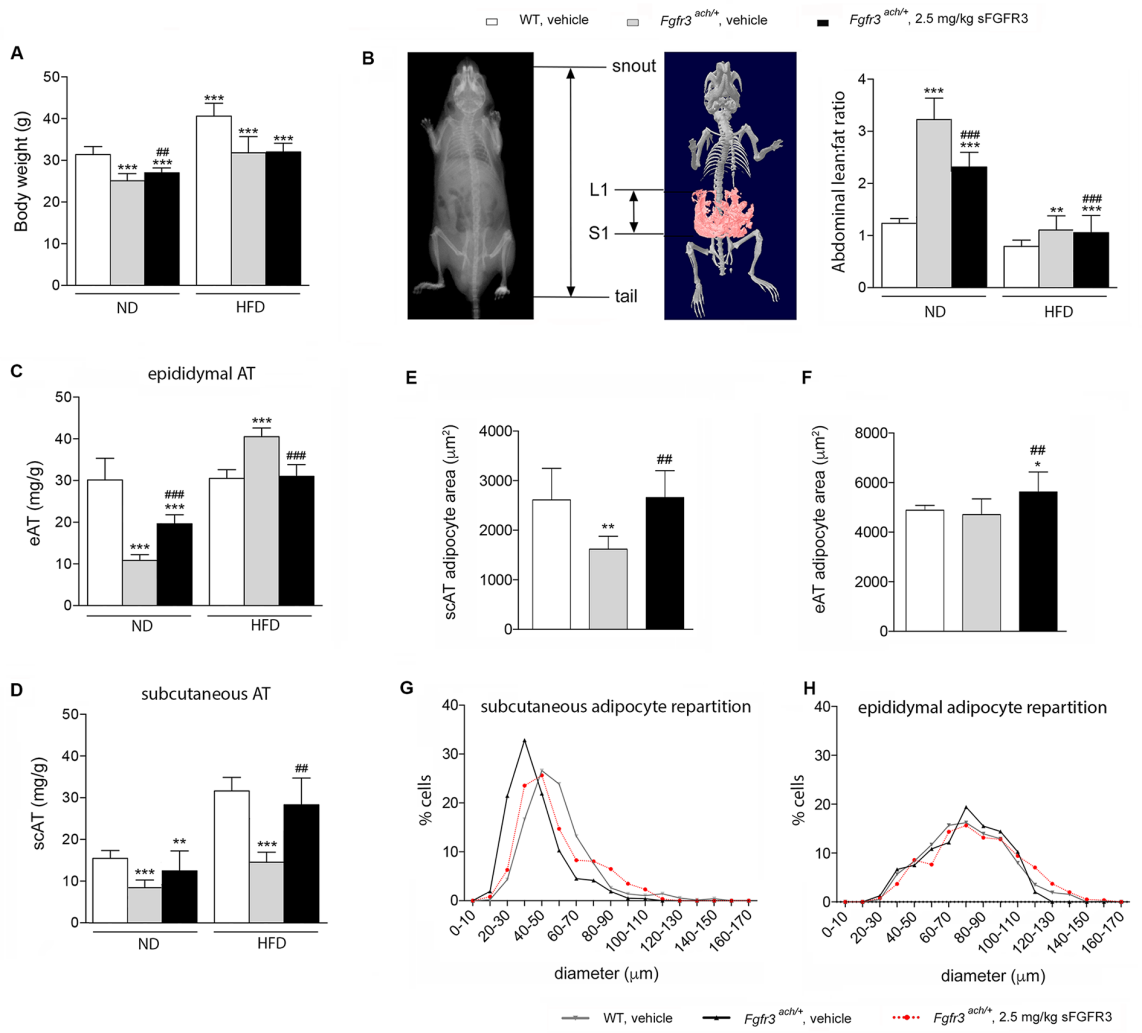
Transgenic *Fgfr3*^*ach/+*^ mice preferentially develop visceral obesity that is prevented upon sFGFR3 treatment. (**A**), body weight of vehicle-treated WT and *Fgfr3*^*ach/+*^ mice and sFGFR3 treated *Fgfr3*^*ach/+*^ mice after 10 weeks of ND or HFD challenge. (**B**), abdominal lean:fat ratio. (**C**) epididymal adipose tissue (eAT) weight and (**D**) subcutaneous adipose tissue (scAT) weight per g of body weight. (**E**) scAT and (**F**) eAT adipocyte area (μm^2^). (**G**) scAT and (**H**) eAT scattering of adipocytes according to their diameter. Data are represented as mean ± SD (n = 8–10 mice to each group). Data followed normal distribution. *p<0.05, **p<0.01, ***p<0.001 versus vehicle-treated WT, ^#^*p*<0.05, ^##^*p*<0.01 versus vehicle-treated *Fgfr3*^*ach/+*^; Student’s *t* test.

After 10 weeks of diet challenge, all HFD groups showed a significant increase in body weight compared to ND (Fig 2A). Interestingly however body composition was different in both genotypes. Untreated *Fgfr3*^*ach*/+^ mice had an higher abdominal lean:fat ratio, measured between L1 and S1, compared to WT mice, independently of the diet (Fig 2B). When fed with ND, untreated *Fgfr3*^*ach*/+^ mice had less epididymal (visceral) and subcutaneous adipose tissues than WT animals (Fig 2C and 2D). However, after 10 weeks of HFD challenge, untreated *Fgfr3*^*ach*/+^ mice developed more epididymal adipose tissue compared to WT animals that preferentially developed subcutaneous fat depot (Fig 2C and 2D). These data showed that, like achondroplasia patients, *Fgfr3*^*ach*/+^ mice were prone to developed visceral adipose tissue.

sFGFR3 treatment has no effect on body weight gain (Fig 2A). However, body composition was significantly impacted by sFGFR3 treatment with a significant decrease in abdominal lean:fat ratio in mice fed with ND (Fig 2B), caused by a decrease in lean masses and an increase in fat masses respectively. Very interestingly, sFGFR3-treated *Fgfr3*^*ach*/+^ mice displayed fat depot distributions that were like those of WT animals whether they were fed with ND or HFD (Fig 2C and 2D).

After the HFD challenge, histological analyses showed smaller adipocytes in the subcutaneous area and a greater proportion of these small adipocytes in untreated *Fgfr3*^*ach*/+^ mice compared to WT (Fig 2E and 2G). No difference in size or dispersion was observed in epididymal adipocytes between both genotypes (Fig 2F and 2H). sFGFR3 treatment restored the subcutaneous adipocytes size and scattering and induced a slight increase in epididymal adipocyte size (Fig 2E–2H). Because increased proportion of small adipose cells has been associated with inflammation [16, 17], several circulating adipokines were measured to evaluate the extent of systemic inflammation in these animals (S1 Fig). Adipokines were sorted into four categories–pro-inflammatory, obesity-related, insulin-pathway and FGFs–all of which were increased in transgenic mice compared to WT littermates (Table 1). Untreated *Fgfr3*^*ach*/+^ mice displayed a low-grade inflammatory baseline compared to WT animals, that was exacerbated under HFD challenge (Table 1). Treated Fgfr3^ach/+^ animals under ND or HFD has a systemic profile that resembled that of their WT littermates.

**Table 1:** Untreated *Fgfr3*^*ach/+*^ mice displayed an elevated inflammatory baseline prevented by sFGFR3 treatment.

	ND			HFD		
	WT	*Fgfr3* ^*ach/+*^		WT	*Fgfr3* ^*ach/+*^	
markers (n)	vehicle	vehicle	2.5 mg/kg sFGFR3	vehicle	vehicle	2.5 mg/kg sFGFR3
**Pro-inflammatory (14)**	**+**	**++**	**+**	**+**	**++**	**+**
**Obesity (15)**	**++**	**+++**	**+**	**++**	**+++**	**++**
**Insulin pathway (7)**	**++**	**++**	**++**	**++**	**+++**	**++**
**FGF (2)**	**+**	**+**	**-**	**-**	**++**	**-**

Pro-inflammatory, obesity, insulin pathway and FGF circulating adipokines expression performed into vehicle-treated WT and *Fgfr3*^*ach/+*^ mice and sFGFR3 treated *Fgfr3*^*ach/+*^ after 10 weeks of ND or HFD challenge. ‘-’ = <2 arbitrary units (A.U.), ‘+’ = 10–30 A.U., ‘++’ = 30–100 A.U., ‘+++’ >100 A.U.

**Fig 3:**
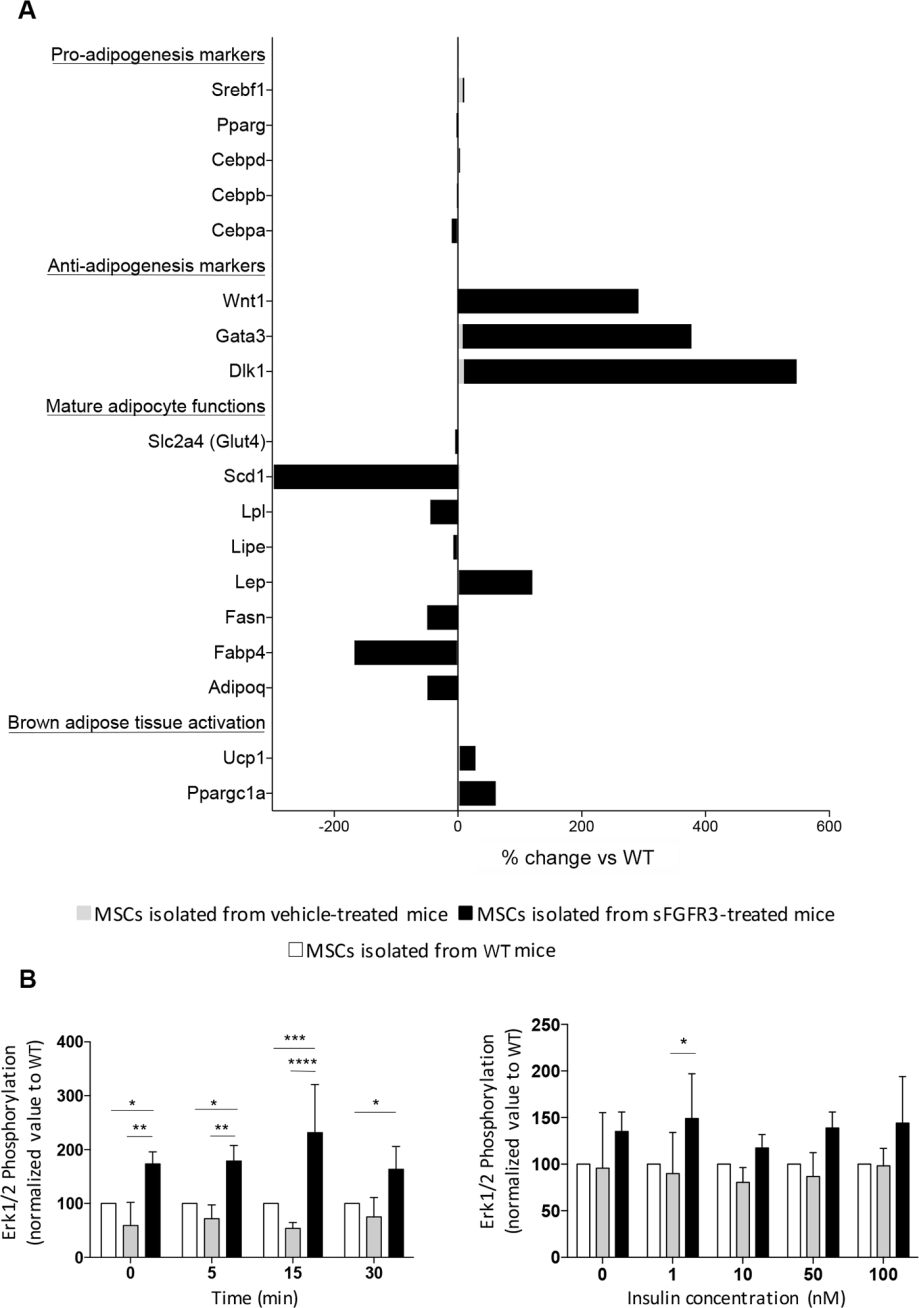
MSCs isolated from untreated or sFGFR3-treated *Fgfr3*^*ach/+*^ mice show pre-engagement towards adipogenesis with no alteration of the insulin response compared to WT mice. **(A)** Expression of genes involved in different steps of adipogenesis differentiation (Genes are listed in **S2 Table**). Expression was normalized to HPRT, RPL6 and RPL13a expression and expressed as percent of change compared to WT. (**B**) Cells were stimulated with 50nM of insulin for 0, 5, 15 or 30 min or with 0, 1, 10, 50 or 100nM of insulin during 5 min. P-Erk1/2 expression, normalized to Erk1/2 total expression, was expressed as normalized value to WT. Data are represented as mean ± SD. Data followed normal distribution. *p<0.05, **p<0.01, ***p<0.001 versus vehicle-treated WT, ^#^*p*<0.05, ^##^*p*<0.01 versus vehicle-treated *Fgfr3*^*ach/+*^; Two-way ANOVA with Tukey’s multiple test.

In vitro, mesenchymal stem cells (MSCs) isolated from *Fgfr3*^*ach/+*^ mice showed that early and intermediary genes of the differentiation process such as Srebf-1, CEBP/d, CEPB/a and PPARg were already expressed (Fig 3A). Very interestingly, MSCs isolated from sFGFR3-treated *Fgfr3*^*ach/+*^ mice showed significant increase in anti-adipogenic markers and brow tissue activation markers as well as decreased expression of genes involved in the functions of mature adipocytes (Fig 3A). Together with the in vivo data, this suggests a predisposition to adipogenesis in *Fgfr3*^*ach/+*^ mice that can be prevented by sFGFR3 treatment.

**Fig 4:**
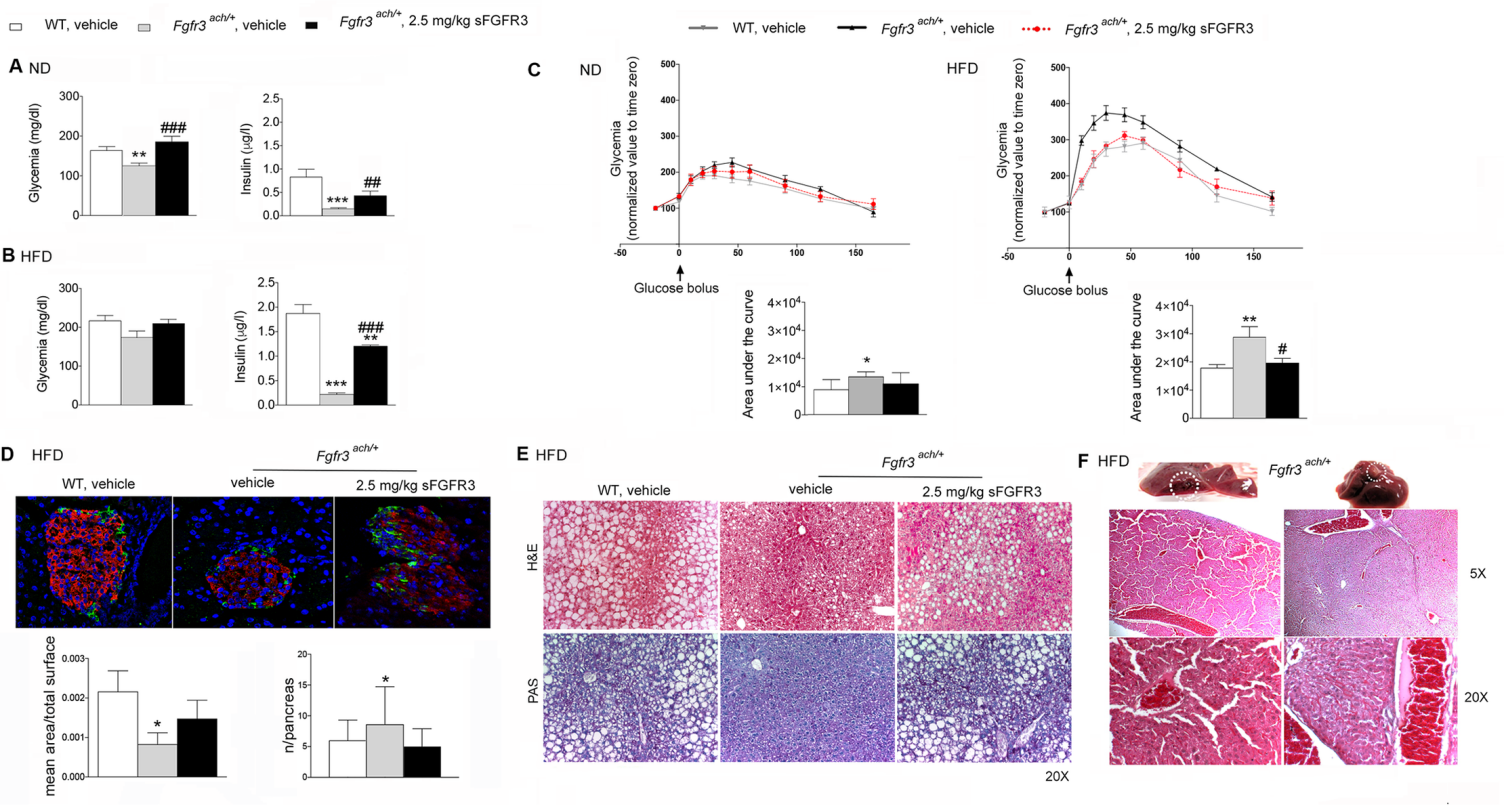
Glucose metabolism is altered in transgenic *Fgfr3*^*ach/+*^ mice and restored with sFGFR3 treatment. Fasting glycemia and insulinemia of mice following 10 weeks of (**A**) ND or (**B**) HFD. (**C**) glucose tolerance test; glucose levels were normalized to the value of time -15 min and area under the curve corresponding to each group of mice. (**D**) Mice pancreas insulin content (immunohistochemistry of paraffin-embedded sections, red: insulin; green: glucose; blue: DAPI staining), mean of pancreas islets normalized to total surface and mean of islets number in each group under HFD condition. (**E**) Liver H&E and PAS staining under HFD condition. (**F**) H&E staining of hepatic nodules. Data are represented as mean ± SD (n = 8–10 mice to each group). Data followed normal distribution. **p<0.01, ***p<0.001 versus vehicle-treated WT, ^#^*p*<0.05, ^##^*p*<0.01 versus vehicle-treated *Fgfr3*^*ach/+*^; Student’s *t* test.

Compared to their WT littermates, mice carrying the FGFR3 mutation had low fasting glycemia and very low baseline levels of insulin (Fig 4A). When challenged with a HFD diet (Fig 4B), glycemic levels raised but remained under those of the WT animals. Insulin levels remained extremely low. In sFGFR3-treated *Fgfr3*^*ach*/+^ animals, glycemia was restored and insulin levels significantly increased compared to untreated Fgfr3^ach/+^mice (Fig 4A and 4B). To evaluate the development of glucose intolerance, glucose tolerance tests (GTT) were performed after 10 weeks of diet challenge. Untreated *Fgfr3*^*ach*/+^ mice displayed higher glucose levels and AUC compared to their WT littermates (Cmax 320.9 ± 32.0 mg/dL and 273.3 ± 23.9 mg/dL, AUC 1.2x10^4^ ± 0.7x10^4^ and 1.7x10^4^ ± 0.4x10^4^, respectively), showing some basal glucose intolerance even under ND. This was further exacerbated under HFD (Fig 4C). When *Fgfr3*^*ach*/+^ mice were treated with 2.5 mg/kg sFGFR3, normal GTT responses were restored (Fig 4C). We attempted to perform insulin tolerance test in transgenic mice under ND or HFD but, because of their lower basal glycemic levels, *Fgfr3*^*ach*/+^ mice did not support insulin injection and died rapidly. Analysis of insulin sensitivity of mesenchymal stem cells isolated from *Fgfr3*^*ach*/+^ or WT mice showed no differences in Erk1/2 phosphorylation levels suggesting similar response to insulin stimulation in both type of mice (Fig 3B). These results suggest that *Fgfr3*^*ach*/+^ mice do not appear more sensitive to insulin regulation but that insulin injection during the ITT probably induced lethal hypoglycemia because of their low basal glycemia. Pancreas analyses showed smaller and more islets of Langerhans with lower insulin and glucagon contents in untreated *Fgfr3*^*ach*/+^ mice (Fig 4D) suggesting an alteration of insulin production and/or storage. This was partially restored in treated animals (Figs 3B and 4A–4C). Glucose storage also appeared impaired in the liver of untreated *Fgfr3*^*ach*/+^ animals as seen by the decrease in glycogen in liver sections (Fig 4E). As expected, following 10 weeks of HFD challenge, WT mice developed grade III macrovesicular steatosis with more than 75% of hepatocytes displayed lipid vacuoles that were larger than the nucleus (Fig 4E). In contrast, after 10 weeks of HFD, untreated *Fgfr3*^*ach*/+^ mice developed reversible benign hepatic nodules (Fig 4F) and a grade II microvesicular steatosis: less than 50% of hepatocytes displayed small vacuoles (Fig 4E). Interestingly, sFGFR3 treatment restored a normal hepatic response in treated *Fgfr3*^*ach*/+^ mice (Fig 4E) and no nodules were observed. Normal liver and pancreas histology are shown for reference in S2 Fig.

**Fig 5:**
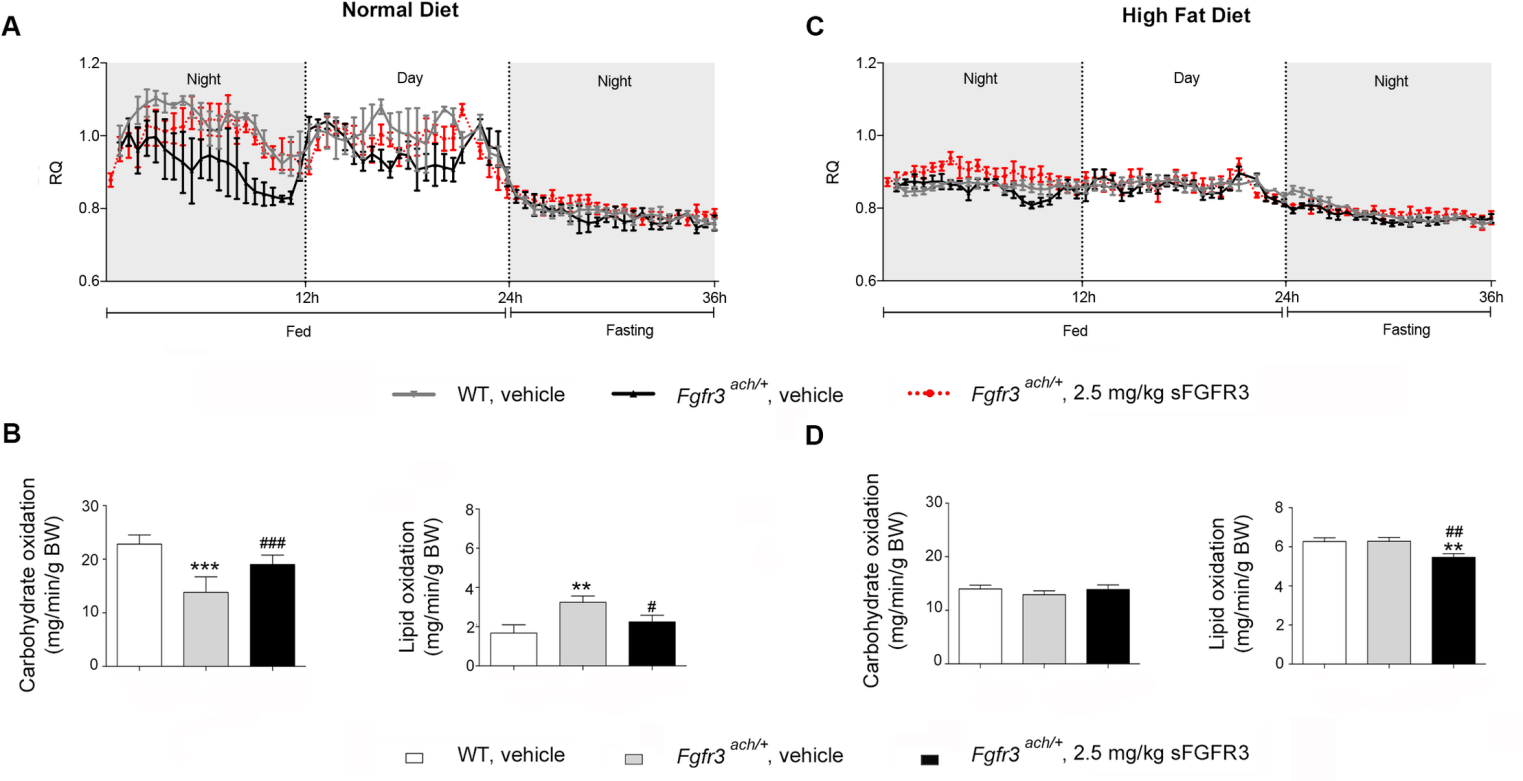
Untreated transgenic *Fgfr3*^*ach/+*^ mice draw essentially their energy from lipids. Basal respiratory quotient (RQ = VCO_2_/VO_2_) during night or day fasting and feeding periods following 10 weeks of ND (**A**) or HFD (**C**) challenge. Basal carbohydrate and lipid oxidation in WT and *Fgfr3*^*ach/+*^ ND (**B**) or HFD (**D**) challenged mice. Data are represented as mean ± SD (n = 8–10 mice to each group). Data followed normal distribution. **p<0.01, ***p<0.001 versus vehicle-treated WT, ^#^*p*<0.05, ^##^*p*<0.01 versus vehicle-treated *Fgfr3*^*ach/+*^; Student’s *t* test.

The basal energy metabolic rate of *Fgfr3*^*ach*/+^ mice was evaluated by indirect calorimetry. We found that while lean WT animals fed with normal diet (ND) drew energy from carbohydrate sources (respiratory quotient RQ near 1), fed transgenic achondroplasia mice drew their energy essentially from lipid sources (RQ near 0.7) (Fig 5A). In fasting episode, as expected, both types of animals drew their energy from lipid sources. This preferential lipid utilization was confirmed by the calculation of carbohydrate and lipid oxidation, which were respectively lower and higher in *Fgfr3*^*ach*/+^ mice compared to WT animals (Fig 5B). Over a 24-hour period, *Fgfr3*^*ach*/+^ mice tend to eat constantly not only during the nocturnal period, however energy expenditure and food intake were not significantly different between both genotypes (S3A–S3D Fig). As expected under HFD, all animals drew their energy from lipid sources, leading to similar carbohydrate and lipid oxidation indexes (Fig 5C and 5D and S2E–S2H Fig). Very interestingly, *Fgfr3*^*ach*/+^ animals that received sFGFR3 treatment during the growth period behaved like untreated WT mice after weaning whether they were fed with ND or HFD, suggesting the restoration of glucose metabolism capacities during the treatment period (Fig 5 and S3 Fig).

## Discussion

Obesity has long been recognized as a common complication of achondroplasia, severely aggravating sleep apnea and musculoskeletal dysfunctions [18]. However, there are no reports in the literature unfolding mechanisms leading to these metabolic disturbances. For many years, overweight and obesity have been simply associated with excess of food intake and/or decreased physical activity [18]. Additionally, this disease is not associated with an increase in typical risks associated with obesity such as type 2 diabetes. Considering obesity as part of the management of achondroplasia is thus not always a priority and certainly not part of standardized care [1]. Obesity is also somewhat difficult to study using classical tools such as BMI or skin fold measurements even though they have a good correlation with fat mass, even in children [18–20]. Indeed, because it is based on height, BMI overestimates body fatness in populations with disproportionate skeletal dysplasia and cannot simply apply in this patient population [21]. To help studying obesity, Hoover-Fong et al. have established achondroplasia-specific BMI charts allowing to reinforce their surveillance implementing nutritional counseling when needed [10]. It seems essential to also assess variations in adipose tissue deposition in the different body sites to evaluate the severity of obesity in achondroplasia. Compared to BMI z-scores and skinfold measurements, the android:gynoid ratio is clearly the most closely related to all risk factors in overweight and obese children [22–24]. In achondroplasia, children display higher android:gynoid ratio that develops very early during childhood. Together with our findings in *Fgfr3*^*ach/+*^ mice, this strongly suggests a predisposition to preferential visceral obesity. Indeed, cells derived from the mesenchymal lineage appeared more prone to adipogenesis than cells isolated from WT animals and seem pre-engaged in the differentiation process towards adipocytes. Moreover, different adipocyte distribution was observed with a greater proportion of small adipocytes in the subcutaneous adipose tissue of *Fgfr3*^*ach/+*^ mice compared to WT animals. Spalding et al. demonstrated that the number of fat cells is set during childhood and adolescence and remains constant during adulthood [25]. Despite modifications in fat mass secondary to obesity, no variation in adipocyte number is observed in adults, and adipose tissue adaptation only depends on adipocyte size change like hypertrophy. If this can translate into achondroplasia, this would suggest that obesity in achondroplasia patients is set very early during childhood and that it would be necessary to include its monitoring as early as 4–8 years old using DXA scans for example.

The development of an abdominal obesity is usually considered to be the most deleterious type of obesity [26]. However, in these patients, it does not correlate with an increase of typical risk factors such as high glucose or high lipid levels. Our results strongly suggest that these observations are a consequence of the mutation affecting FGFR3. Currently, three members of the FGFs family have been linked to obesity [27]: FGF1, FGF15/19 and FGF21. FGF1 is regulated by PPARg, and is notably highly upregulated in WAT [28]. FGF1 is known to promote pre-adipocyte proliferation and differentiation through Erk1/2 signaling. It also triggers acute blood lowering effect that seem to be dependent on FGFR2 signaling in WAT. FGF15/19 is considered as a regulator of the feeding responses. It binds to FGFR4/ßklotho receptor complex on the cell membrane of hepatocytes ultimately leading to repression of gluconeogenesis [29]. FGF21 mainly binds to FGFR1 and regulates the adaptive fasting response through PPARa [30]. In *Fgfr3*^*ach/+*^ mice, overexpression of FGFR3ach during embryogenesis could modify FGFs signaling in different cell types. In accordance with this, we found that mesenchymal stem cells from *Fgfr3*^*ach/+*^ mice express high levels of FGFR3 compared to their WT littermates (9-fold increase). Similarly, newborn *Fgfr3*^*ach/+*^ mice express increased levels of FGFR2 and FGFR4 in AT and liver (S4 Table). Altogether these data could explain the low glycemia associated with abdominal obesity in *Fgfr3*^*ach/+*^ mice. Along this line, we also observed that patients even tended to lower fasting glycemia and insulinemia and no patients had glucose intolerance, suggesting that similar mechanisms could apply in human. sFGFR3 treatment was applied immediately after birth and prevented most metabolic complications, including the development of abdominal obesity. Treated *Fgfr3*^*ach/+*^ mice were not protected against obesity *per se* but behave essentially like WT animals with the development of homogeneous obesity and the restoration of glucose metabolism leading to glucose resistance under HFD. This suggests that, if apply early in life, treatment could revert the effect of the Fgfr3ach mutation on these atypical metabolic tissues.

Although other innovative treatments have been described and are in development, they do not describe or discuss potential impact on the development of achondroplasia obesity [1]. Statins have been used in a repositioning strategy and show interesting effects on the restoration of skeletal bone growth in achondroplasia [31]. However, achondroplasia children already show levels of cholesterol that are in the low range and the chronic use of this lipid-lowering medication may not be indicated. Vosoritide, an analog of C-type natriuretic peptide (CNP), is currently in phase 2 clinical trials effectively restoring bone growth in children with achondroplasia [32]. CNP is part of the NPs family, comprising three members—atrial NP (ANP), brain-type NP (BNP) and CNP—that are considered to play a role in metabolic regulation, improving lipid mobilization, oxidative metabolism and blood pressure [33]. Although NPs could potentially have effect on classical obesity, it remains uncertain whether a CNP analog could be beneficial to prevent obesity in achondroplasia in view of the low lipid profiles seen in these patients.

### Conclusions

In conclusion, BMI and precise growth curves have been established for children with achondroplasia for several decades [10, 18, 34, 35]. Our data are in accordance with them but also establish that the development of a nonconforming obesity is preferentially abdominal and appears to be triggered by the FGFR3 mutation. Even though there is no correlation with increased risk of diabetes, it is necessary to monitor the development of obesity in these patients as it leads to increased severe co-morbidities and CVS risks. This study also shows that, if administered early in life, sFGFR3 appears to be a promising treatment for achondroplasia, targeting together bone growth and preventing the development of this atypical obesity.

## Materials and methods

### Clinical analysis

To evaluate the potential of sFGFR3 therapy to prevent the development of abdominal obesity in achondroplasia, we have first characterized the development of obesity in children by doing a retrospective chart review. For this, eleven subjects (5 girls and 6 boys) with achondroplasia were followed from birth up to 18 years in the Department of endocrinology, bone diseases, genetic and medical gynecology of the Purpan Children’s Hospital in Toulouse, France. This retrospective chart review study was conducted in accordance with the declaration of Helsinki and the French regulations on Biomedical research (Jarde law, décret n° 2016–1537 du 16 novembre 2016), not requiring ethics committee approval for a non-interventional study. Data were fully anonymized and the study was declared to the CNIL (Commission Nationale de l’Informatique et des Libertés, Chair of the National Data Protection Commission) (ref # 2127581).

All patients harbored the G380R FGFR3 mutation, confirmed by molecular testing. Patients receiving any growth treatment were excluded. Children were followed in the specialized center every 4 months on average. During each visit, data included the height, weight and subsequent body mass index (BMI = kg/m^2^). Blood analyses were performed only once a year and dual-energy X-ray absorptiometry (DXA) was performed once.

Anthropometric calculations, including height-to-age z score and BMI-to-age z score, were done using the WHO AnthroPlus software (WHO AnthroPlus for personal computers Manual: Software for assessing growth of the world's children and adolescents. Geneva: WHO, 2009 (http://www.who.int/growthref/tools/en/); z-scores are based on WHO standards (birth to 60 months) and WHO reference 2007 (61 months to 19 years). Body composition was evaluated by DXA using the Lunar Prodigy device (GE Healthcare). The regions of interest (ROI) for regional body composition were defined using the manufacturer’s instructions (**Fig 1B**). Briefly, the trunk ROI was measured from the pelvis cut (lower boundary) to the neck cut (upper boundary); the android ROI was measured from the pelvis cut (lower boundary) to above the pelvic cut by 20% of the distance between the neck and the pelvis cuts (upper boundary); the umbilicus ROI was defined from the lower boundary of the android by 150% of the android distance; and the gynoid ROI was from the lower boundary of the umbilicus ROI to a line equal to twice the height of the android ROI (lower boundary).

Blood samples were drawn after at least a 12-hour fasting period and analyzed at the Federative Institute of Biology (IFB) of the Purpan hospital. Fasting glucose and insulin, total, HDL, and LDL cholesterol, triglyceride, TGO, TGP, gGT concentrations, as well as plasma total calcium, sodium, potassium, bicarbonate, phosphate, chloride and alkaline phosphatase were measured using standard colorimetric or colorimetric enzymatic methods on the Cobas 8000 modular analyzer series, using the C701 module, from Roche Diagnostics. Serum concentration of total 25OH vitamin D was measured by chemiluminescent immunoassay method on the Cobas 8000 modular analyzer series, using the E602 module, from Roche Diagnostics. All values were compared to reference values established for each age group within the Children’s hospital and using published references [36–38].

After 12h overnight fast, OGTT was performed in patients per the established recommendations[39]. Following oral administration of 1.75g/kg of glucose, blood samples were drawn at baseline and after 30 and 120 min to measure glucose concentration using hexokinase method. Glucose regulation was assessed according to the American Diabetes Association guidelines: normal glucose regulation was defined as fasting glucose <5.6 mmol/L and 120 min glucose <7.8 mmol/L and impaired fasting glucose as fasting glucose 5.6–6.9 mmol/L, impaired glucose tolerance as 120 min glucose 7.8–11.0 mmol/L.

### Animals and treatment

Experiments were performed on transgenic *Fgfr3*^*ach/+*^ mice, a mouse model recapitulating most human symptoms. [40]. The study was performed on transgenic *Fgfr3*^*ach/+*^ mice or their wild-type (WT) littermates. For this, litters were treated blindly twice weekly by subcutaneous injections of 2.5 mg/kg of sFGFR3 or vehicle starting at day 3 until day 21. Mice were weaned at age 3 weeks. After one week of acclimation, mice were challenged with normal (ND) or high-fat diet (HFD) for 10 weeks. The development of obesity was evaluated through measures of body composition, indirect calorimetry and classical evaluation of glucose and lipid profiles, as well as hepatic and pancreatic function evaluations. The n per group is presented in the figure legends. At weaning, ear biopsies were used to verify mice genotype by PCR of genomic DNA as previously described [6]. During the experiment, mice were housed in standard laboratory conditions and were allowed access food and water ad libitum. The study was approved by the local Institutional Ethic Committee for the use of Laboratory Animals (CIEPAL Azur) (approvals # NCE-2012-52 and NCE-2015-225). Young *Fgfr3*^*ach/+*^ mice can develop potential complications of achondroplasia. As such, during the first three weeks of life, mice were individually observed daily. Notably, investigators were looking carefully for the onset of bilateral paralysis or breathing problems. If an animal presented some difficulty breathing or paralysis, as seen by the presence of bladder dysfunction, it was euthanized immediately. After weaning, the development of complications is very rare and the phenotype is not harmful anymore. Animals were still observed every two days and any modification of behavior (such as prostration or absence of grooming) lead to the immediate euthanasia of the animal. When appropriate, animals were sacrificed by lethal intraperitoneal injection of pentobarbital.

At day 3, newborn mice were treated with 2.5 mg/kg of FLAG-tagged sFGFR3 as described previously [6]. Control litters received 10 μl of PBS containing 50% glycerol (vehicle). From age day 3 to day 22, *Fgfr3*^*ach/+*^ mice received 6 subcutaneous injections of sFGFR3 or vehicle. One week after weaning at 4 weeks of age, treated and untreated mice were divided into two groups and challenged for 10 weeks with normal (ND, A03, SAFE) or high fat diet (HFD, 52% kcal as fat, custom made, containing 54% lipids, SAFE), respectively.

After 6 hours fasting, blood was taken from the tail vein. Glycemia was measured with a glucometer (Abbot) and serum insulin contents were determined by ELISA (Mercodia). Glucose tolerance tests (GTT) were performed on mice after 10 weeks of ND or HFD challenge. After 6 hours fasting mice were injected with an intra-peritoneal glucose solution (1g/kg). Blood was taken from the tail vein and glucose levels were monitored over time using a glucometer or using EnzyChrom Glucose Assay Kit (BioAssay Systems). Glucose levels were normalized to the value of time -15 min of each mouse.

Indirect calorimetry was studied on mice challenged for 10 weeks with normal (ND) or high fat (HFD) diet. As stated above, mice were treated during the growth period with 2.5 mg/kg of sFGFR3 or vehicle, were subjected to the diet challenge for 2 weeks and were then subjected to the metabolic chambers. After 24 h of acclimatization in individual metabolic cages, O_2_ consumption (VO_2_) and CO_2_ production (VCO_2_) were measured (Oxylet; Panlab-Bioseb) in individual mice at 32 min intervals during a 24 h period with unrestricted access on food followed by one night fasted. The respiratory quotient was calculated and analyzed as follows: RQ = VCO_2_/VO_2_, RQ = 1 correspond to carbohydrate oxidation and RQ~0.7 correspond to fat oxydation. Energy expenditure (kcal/day/weighx0.75 = 1.44xVO_2_x[3.815+1.232xRQ]), carbohydrate (g/min/kg0.75 = [4.55xVCO_2_]-[3.21xVO_2_]) and lipid (g/min/kg0.75 = [1.67xVO_2_]- [1.67xVCO_2_]) oxidations were calculated. Ambulatory activities and rearing of the mice were monitored by a weight transducer technology or an infrared photocell beam interruption method (Oxylet; Panlab-Bioseb).

Body composition was determined using a SkyScan 1178 X-ray micro-CT system. Four and 10 weeks old mice were anesthetized and scanned using the same parameters: 104 μm of pixel size, 49 kV, 0.5 mm thick aluminum filter, 0.9° of rotation step. Total adipose tissue volume was determined between the tip of the snout and the top of the tail and abdominal adipose tissue volume was determined between the lumbar L1 and the sacral S1. Then, adipose tissue quantification was carried out more precisely. Body composition analysis is based on the delimitation of region of interest after 3D reconstruction of scanned images. 3-D reconstructions analyses were performed using NRecon and CTAn software (Skyscan).

At sacrifice, animals were weighted and several tissues and organs (subcutaneous, epididymal adipose tissue, liver, pancreas) were harvested for further analysis by histochemistry or qPCR. In some groups, bone-marrow-derived mesenchymal stem cells were harvested by flushing the femurs [41].

Lipid profile was evaluated by taking intracardiac blood. Total cholesterol, triglycerides (TG), High Density Lipoprotein (HDL) and Low Density Lipoprotein (LDL) were measured on serum using a Beckman AU 2700 Analyzer. Sera were analyzed for protein levels of selected adipokines related to inflammation, obesity, insulin pathway or FGFs using the Mouse Adipokine Array (#ARY013, R&D Systems) according to the manufacturer’s instructions on nitrocellulose membranes. Following streptavidin-HRP and chemiluminescent detection the proteins bound to each captured antibody were quantified using densitometry and levels were compared to percent change from WT mice.

### Mesenchymal stem cell studies

Mesenchymal stem cells were isolated from femoral bone marrow providing from untreated or sFGFR3-treated 6 to 8 weeks old mice by flushing the femurs [41] and cultivated to 80% confluence in medium supplemented with 10% serum. Then, medium was replaced with adipogenic medium (DMEM F12 supplemented with 2% serum, 1% antiobiotics, 66mM insulin, 1nM triiodothyronine, 100mM cortisol, 10μg/ml transferrin and 3μM rosiglitazone in DMEM-F12). Total RNAs were extracted using Trizol Reagent (Life Technologies) and Chloroform (Sigma). One microgram of total RNA was reverse transcribed into cDNA using random hexamers and the Superscript II Reverse Transcriptase kit (Invitrogen). Real-time qPCR was performed on a StepOne Plus Real-Time PCR System (Applied Biosystems) with Fast SYBR Green Master Mix (Sigma) using a custom RT2 Profiler PCR Array (CAPM13080). Studied genes are listed in **S5 Table.** Gene expression was normalized to HPRT, RPL13a and RPL6 housekeeper gene.

FGFRs expression was performed using the following primers: FGFR1, For-5' CAGATGCACTCCCATCCTCG 3' Rev-5' TCT GGGGATGTCCAGTAGGG 3'; FGFR2 For-5' TGGCAGTGAAGATGTTGAAAG 3' Rev-5' ATCATCTTCATCATCTCCATCTCTTG 3'; FGFR3 For-5' TTATCCTTGGCTCCTGGGTG 3' Rev-5' CTGGAAGGTAGCAGTGGGAA 3'; FGFR4 For-5' GCTCGGAGGTAGAGGTCTTGT 3' Rev-5' CCACGCTGACTGGTAGGAA 3'; HSP90 For-5' TTTGGTGGACACAGGCATTG 3' Rev-5' CAAACTGCCCGATCATGGAG 3'.

For insulin signaling experiments, 24h after isolation, cells were depleted during 6h and then stimulated with 50nM insulin for 0, 5, 15, 30 min, or with 0, 1, 10, 50, 100nM for 5 min. Cells extracts were processed for Western Blot analysis. For this, cells were lysed in RIPA lysis buffer (Millipore) and homogenized from 30 min using a vortex. Proteins were pelleted by centrifugation 1400 g from 15 min and total protein contents were evaluated using the BCA Protein Assay Kit (ThermoScientific). Samples were diluted in Sample Reducing Buffer (Life Technology), boiled, and processed for immunoblotting by using a standard procedure. Monoclonal antibodies were used as follows: anti p44/42 MAPK (Erk1/2) (4695S, Cell Signaling), anti phospho-p44/42 MAPK (Erk1/2) (Thr202/Tyr204) (4370S, Cell Signaling). Results were normalized to HSP90 expression (4877S, Cell Signaling).

### Histology and immunohistochemistry

Histology analyses were performed on adipose tissue, liver and pancreas of 12 weeks old mice. Organs were fixed in 4% formalin for 24 h, paraffin-embedded and 5 μm sections are stained with hematoxylin and eosin. Adipocyte diameters were measured in one or two different sections in each sample (from 100 to 300 adipocytes were counted in each section). Pancreatic islet numbers and area were measured in one section using Fiji Image J system (islet area was normalized to total pancreas area). Liver glycogen content was evaluated by Periodic acid-Shiff (PAS) staining.

Immunohistochemistry was performed on 5μm sections of pancreas. Sections were blocked 45 min with PBS 1% BSA, incubated over night with anti-insulin monoclonal primary antibody (4μg/ml) (Santa Cruz, sc-9168), anti-glucagon polyclonal antibody (4μg/ml) (Santa Cruz, sc-7779) and 1h with Alexa Fluor 594 secondary antibody (2μg/ml) (Life Technologies, A-21442) and Alexa Fluor 488 secondary antibody (4μg/ml) (Life Technologies, A-21467) in wet chamber. Sections were counterstained with DAPI solution (Santa Cruz), treated with autofluorescence eliminator reagent and visualized under fluorescence microscopy. Staining without secondary antibody was used as a negative control.

### Statistical analysis

Statistical analyses were performed with GraphPad Prism 6.0 software. Data were expressed as mean ± SD. To verify normality and equal variance, an Agostino and Pearson omnibus normality test (a = 0.05), a Shapiro-Wilk normality test (a = 0.05) and a KS normality test (a = 0.05) were performed. Significance was determined by using unpaired two-tailed Student *t* test or Tukey’s multiple comparison test. In patients, the raw results of height and BMI were transformed to age-specific z-score from the average in the reference population using reference data. The expected mean result of these values in a healthy population is 0. P values < 0,05 were considered significant (* P< 0,05; ** P< 0,01; *** P< 0,001).

## Supporting information

S1 Fig Circulating adipokines studied in the serum of untreated or sFGFR3-treated *Fgfr3*^*ach/+*^ mice. Results were expressed as percent of change compared to WT. AgRP, agouti-related protein; ANGPT-L3, angiopoietin-3; CRP, C-reactive protein; DPPIV, dipeptidyl peptidase V; FGF, fibroblast growth factor; HGF, hepatocyte growth factor; ICAM-1, intercellular adhesion molecule-1; IGF, insulin-like growth factor; IGFBP, insulin-like growth factor binding protein; MCP-1, monocyte chemotactic protein-1; M-CSF, macrophage colony-stimulating factor; Pref-1, preadipocyte factor 1; RAGE, receptor for advanced glycation endproducts; RANTES, receptor upon activation, normal T-cell expressed and secreted; RBP4, retinol binding protein; TIMP-1, tissue inhibitor of metalloproteinases; VEGF, vascular endothelial growth factor.
(TIF)

S2 Fig Representative histological images of liver (H&E and PAS staining) and pancreas (immunohistochemistry of paraffin-embedded sections, red: insulin; green: glucose; blue: DAPI staining) under ND. (TIF)

S3 Fig Transgenic achondroplasia mice displayed normal energy expenditure, cumulative activity and cumulative rearing during indirect calorimetry. (**A, E**) Basal oxygen consumption, (**B, F**) basal carbon dioxide production, (**C, G**) basal energy expenditure during night or day fasting and feeding periods and (**D, H**) basal cumulative activity and rearing in WT and *Fgfr3*^*ach/+*^ ND and HFD challenged mice, respectively. Data are represented as mean ± SD (n = 8–10 mice to each group). Data followed normal distribution. **p<0.01, ***p<0.001 versus vehicle-treated WT, ^###^*p*<0.001 versus vehicle-treated *Fgfr3*^*ach/+*^; Student’s *t* test.
(TIF)

S1 Table Weight, height and BMI measurements in the three age groups based on sex/ No statistical differences were observed. (DOCX)

S2 Table Densitometry results of achondroplasia patients in the three age groups. (DOCX)

S3 Table Blood parameters in the different age groups. No statistical differences were observed between the groups.
(DOCX)

S4 Table Expression of FGFRs 1–4 in subcutaneous whit adipose tissue (scWAT), brown adipose tissue (BAT), pancreas and liver in 3 days old *Fgfr3*^*ach/+*^ pups. (DOCX)

S5 Table List of genes studied using custom RT2 Profiler PCR Array in mesenchymal stem cells. (DOCX)
