# Supplementary figures and images for: Early postnatal soluble FGFR3 therapy prevents the atypical development of obesity in achondroplasia
Source: PLoS One. 2018 Apr 13;13(4):e0195876. doi: 10.1371/journal.pone.0195876 (PMC5898762; doi:10.1371/journal.pone.0195876)

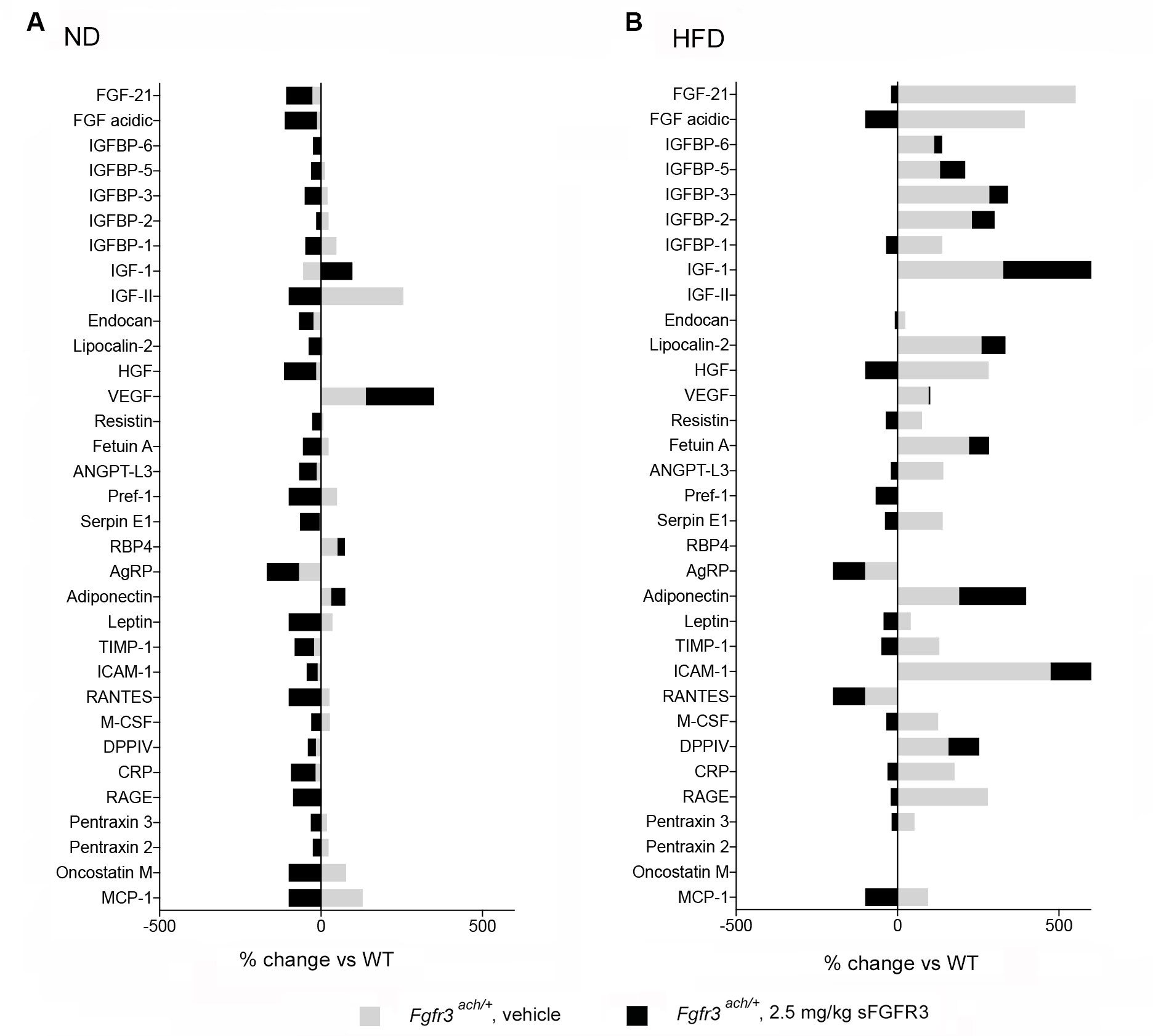

Supplement: S1 Fig — Results were expressed as percent of change compared to WT. AgRP, agouti-related protein; ANGPT-L3, angiopoietin-3; CRP, C-reactive protein; DPPIV, dipeptidyl peptidase V; FGF, fibroblast growth factor; HGF, hepatocyte growth factor; ICAM-1, intercellular adhesion molecule-1; IGF, insulin-like growth factor; IGFBP, insulin-like growth factor binding protein; MCP-1, monocyte chemotactic protein-1; M-CSF, macrophage colony-stimulating factor; Pref-1, preadipocyte factor 1; RAGE, receptor for advanced glycation endproducts; RANTES, receptor upon activation, normal T-cell expressed and secreted; RBP4, retinol binding protein; TIMP-1, tissue inhibitor of metalloproteinases; VEGF, vascular endothelial growth factor. (TIF) [file pone.0195876.s001.tif]

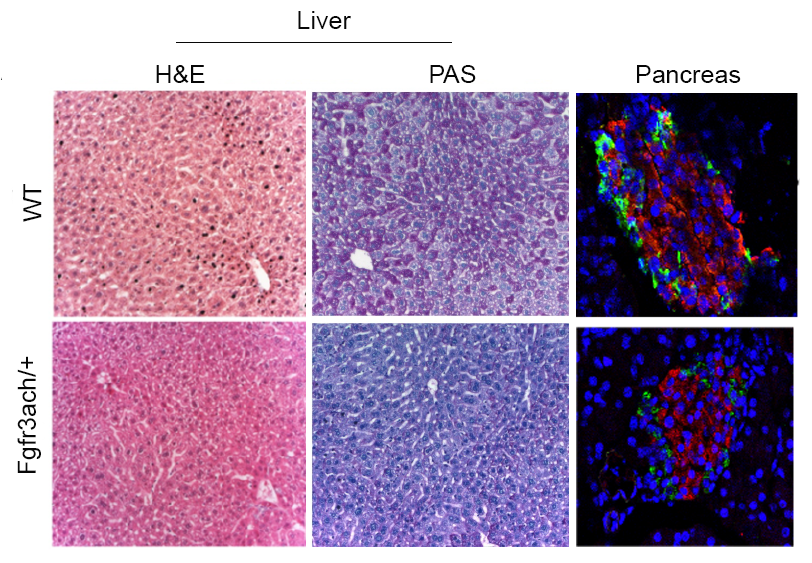

Supplement: S2 Fig — (TIF) [file pone.0195876.s002.tif]

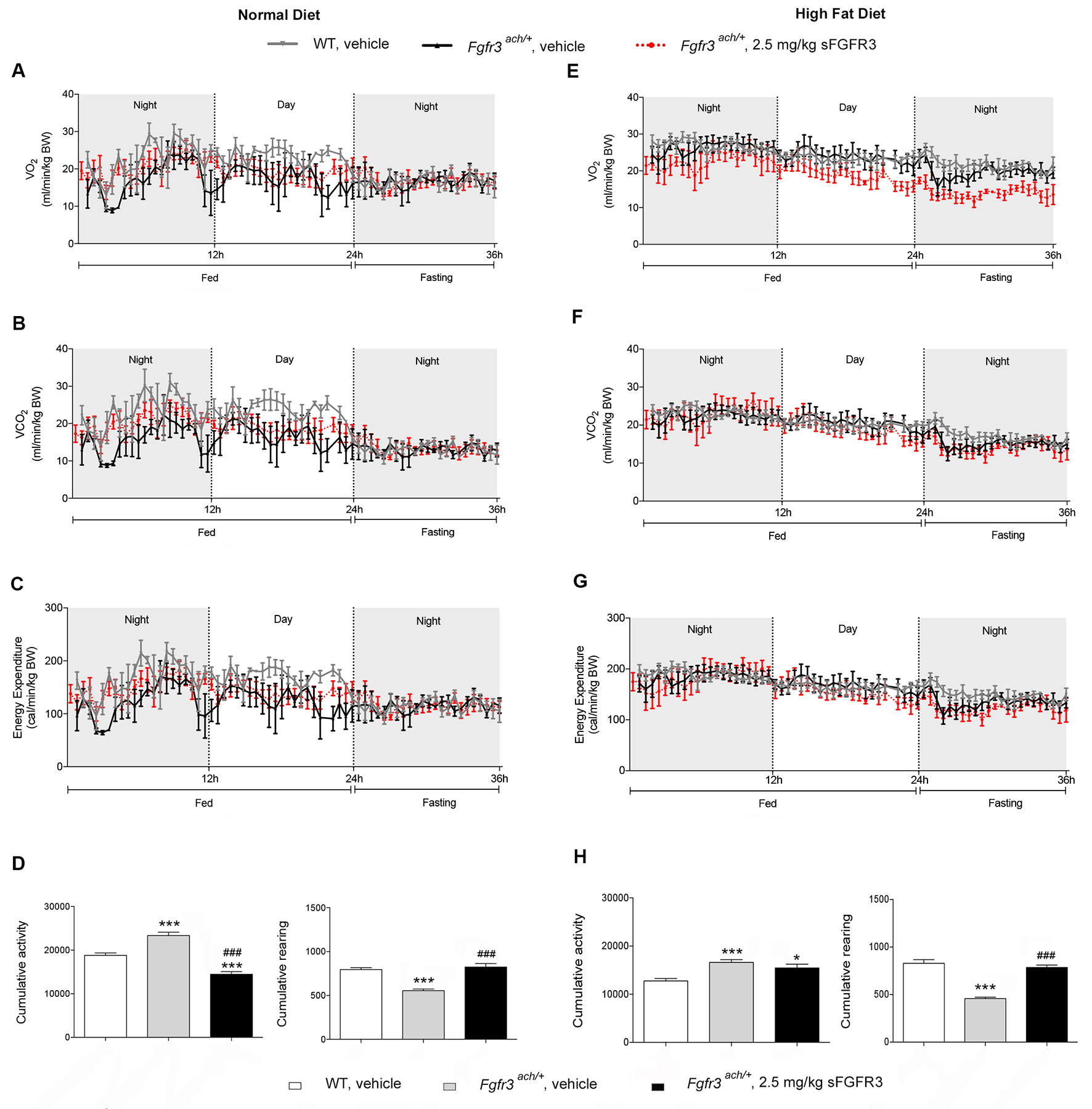

Supplement: S3 Fig — (A, E) Basal oxygen consumption, (B, F) basal carbon dioxide production, (C, G) basal energy expenditure during night or day fasting and feeding periods and (D, H) basal cumulative activity and rearing in WT and Fgfr3ach/+ ND and HFD challenged mice, respectively. Data are represented as mean ± SD (n = 8–10 mice to each group). Data followed normal distribution. **p<0.01, ***p<0.001 versus vehicle-treated WT, ###p<0.001 versus vehicle-treated Fgfr3ach/+; Student’s t test. (TIF) [file pone.0195876.s003.tif]
