# Supplementary material for: Early postnatal soluble FGFR3 therapy prevents the atypical development of obesity in achondroplasia
Source: PLoS One. 2018 Apr 13;13(4):e0195876. doi: 10.1371/journal.pone.0195876 (PMC5898762; doi:10.1371/journal.pone.0195876)
Supplement: S1 Table — (DOCX) [file pone.0195876.s004.docx]

**S1 Table. Weight, height and BMI measurements in the three age groups based on sex/ No statistical differences were observed.**

| **Age (yrs)** | **Sex** | **Weigth (kg)** | **Height (cm)** | **BMI (kg/m^2^)** |
| --- | --- | --- | --- | --- |
|  |  |  |  |  |
|  |  |  |  |  |
| [0-3] | F | 7.53 ± 2.77 | 63.63 ± 10.42 | 18.03 ± 2.47 |
|  | M | 8.19 ± 2.84 | 64.57 ± 9.04 | 18.88 ± 2.14 |
|  |  |  |  |  |
|  |  |  |  |  |
| [3-8] | F | 17.59 ± 4.79 | 92.55 ± 5.77 | 20.26 ± 3.15 |
|  | M | 19.17 ± 5.20 | 92.09 ± 6.22 | 22.22 ± 3.47 |
|  |  |  |  |  |
|  |  |  |  |  |
| [9-18] | F | 38.90 ± 7.45 | 117.84 ± 6.18 | 27.72 ± 2.60 |
|  | M | 47.07 ± 10.83 | 118.92 ± 5.62 | 32.81 ± 4.98 |
|  |  |  |  |  |
