# Supplementary material for: Early postnatal soluble FGFR3 therapy prevents the atypical development of obesity in achondroplasia
Source: PLoS One. 2018 Apr 13;13(4):e0195876. doi: 10.1371/journal.pone.0195876 (PMC5898762; doi:10.1371/journal.pone.0195876)
Supplement: S2 Table — (DOCX) [file pone.0195876.s005.docx]

**S2 Table. Densitometry results of achondroplasia patients in the three age groups.** Values are mean ± SD (range). The *p* values represent the significance of the difference between the three groups (one way ANOVA test followed by Tukey’s multiple comparison tests, ns = non-significant). Results of post hoc analyses: ^a^ significantly different between [0-3] and [4-8] groups; ^b^ significantly different between [0-3] and [9-18] groups; ^c^ significantly different between [4-8] and [9-18] groups.

| **Age (yrs)** | **[0-3]** | | **[4-8]** | | **[9-18]** | |  | |  |
| --- | --- | --- | --- | --- | --- | --- | --- | --- | --- |
| **Variables** | **Mean ± SD** | **Range (min-max)** | **Mean ± SD** | **Range (min-max)** | **Mean ± SD** | **Range (min-max)** | |  |  |
| **Fat and lean mass repartition (kg)** |  |  |  |  |  |  | | *P* |  |
| Total body fat mass | 1.52 ± 0.32 | (1.29-1.76) | 2.64 ± 0.73 | (1.98-4.06) | 19.78 ± 7.98 | (8.56-30.15) | | < 0.0001 | ^b. c^ |
| Total body lean mass | 7.04 ± 0.97 | (6.35-7.77) | 13.29 ± 1.89 | (9.36-15.32) | 23.13 ± 2.86 | (18.94-26.65) | | 0.001 | ^b, c^ |
| Trunk mass | 3.67 ± 0.45 | (3.38-4.02) | 6.93 ± 1.46 | (4.83-8.68) | 21.19 ± 5.53 | (14.08-28.72) | | < 0.0001 | ^b, c^ |
| Trunk fat mass | 0.41 ± 0.12 | (0.32-0.49) | 0.83 ± 0.44 | (0.23-1.64) | 9.74 ± 4.42 | (3.97-15.65) | | 0.001 | ^b, c^ |
| Trunk lean mass | 3.29 ± 0.33 | (3.06-3.53) | 6.11 ± 1.15 | (4.35-7.08) | 11.4 ± 1.50 | (9.67-13.42) | | < 0.0001 | ^a, b, c^ |
| Leg mass | 1.77 ± 0.28 | (1.57-1.97) | 4.61 ± 1.29 | (3.12-7.21) | 13.89 ± 3.36 | (8.86-18.24) | | < 0.0001 | ^b, c^ |
| Leg fat mass | 0.65 ± 0.12 | (0.57-0.73) | 1.22 ± 0.31 | (0.86-1.69) | 7.46 ± 2.61 | (3.39-10.72) | | < 0.0001 | ^b, c^ |
| Leg lean mass | 1.11 ± 0.16 | (0.99-1.23) | 3.39 ± 1.04 | (2.26-5.52) | 6.31 ± 0.98 | (4.81-7.51) | | < 0.0001 | ^a, b, c^ |
| Arm mass | 0.65 ± 0.04 | (0.62-0.68) | 1.21 ± 0.23 | (0.99-1.53) | 3.74 ± 1.03 | (2.38-5.12) | | < 0.0001 | ^b, c^ |
| Arm fat mass | 0.09 ± 0.06 | (0.08-0.10) | 0.21 ± 0.09 | (0.12-0.35) | 1.57 ± 0.76 | (0.56-2.54) | | 0.004 | ^b, c^ |
| Arm lean mass | 0.56 ± 0.04 | (0.53-0.58) | 0.99 ± 0.18 | (0.66-1.18) | 2.16 ± 0.34 | (1.62-2.65) | | 0.001 | ^b, c^ |
|  |  |  |  |  |  |  | |  |  |
| **Fat and lean mass repartition (% of total body weight)** |  |  |  |  |  |  | |  |  |
| Total body fat mass | 19.04 ± 0.74 | (18.51-19.57) | 16.08 ± 3.10 | (11.86-21.40) | 42.93 ± 9.11 | (28.36-52.89) | | < 0.0001 | ^b, c^ |
| Total body lean mass | 88.29 ± 3.51 | (85.81-90.77) | 83.89 ± 9.25 | (74.75-90.43) | 46.19 ± 0.79 | (45.63-68.49) | | < 0.0001 | ^b, c^ |
| Trunk mass | 46.47 ± 2.57 | (44.64-48.29) | 47.26 ± 4.08 | (28.42-50.15) | 50.35 ± 0.06 | (45.00-50.40) | | ns |  |
| Trunk fat mass | 5.01 ± 0.64 | (4.56-5.46) | 5.60 ± 1.74 | (1.37-8.65) | 27.11 ± 0.50 | (13.16-27.46) | | < 0.0001 | ^b, c^ |
| Trunk lean mass | 41.47 ± 3.22 | (39.19-43.74) | 41.66 ± 5.82 | (27.05-45.78) | 23.24 ± 0.44 | (22.71-33.47) | | 0.004 | ^b, c^ |
| Leg mass | 22.14 ± 0.37 | (21.88-22.40) | 25.22 ± 0.28 | (25.03-42.45) | 31.13 ± 1.24 | (29.68-32.32) | | 0.0285 | ^c^ |
| Leg fat mass | 8.17 ± 0.02 | (8.16-8.19) | 6.94 ± 0.87 | (5.95-9.95) | 18.05 ± 1.08 | (11.23-19.32) | | < 0.0001 | ^b, c^ |
| Leg lean mass | 13.97 ± 0.40 | (13.69-14.26) | 18.28 ± 0.60 | (17.64-32.50) | 13.07 ± 0.16 | (12.96-18.44) | | 0.0122 | ^c^ |
| Arm mass | 8.21 ± 0.88 | (7.59-8.83) | 7.33 ± 0.21 | (6.66-8.07) | 8.89 ± 0.14 | (7.60-8.99) | | 0.0032 | ^c^ |
| Arm fat mass | <0.001 | (10^-6^-10^-5^) | 1.08 ± 0.42 | (0.78-1.88) | 4.40 ± 0.09 | (1.86-4.46) | | < 0.0001 | ^b, c^ |
| Arm lean mass | 7.05 ± 0.76 | (6.51-7.59) | 6.25 ± 0.21 | (5.44 ± 6.60) | 4.49 ± 0.23 | (4.25-6.04) | | 0.0005 | ^b, c^ |
|  |  |  |  |  |  |  | |  |  |
| **Gynoid and android body**  **composition (% total tissue)** | |  |  |  |  |  | |  |  |
| Gynoid body fat | 43.75 ± 0.07 | (43.70-43.80) | 33.97 ± 3.00 | (29.30-38.40) | 52.91 ± 6.37 | (40.60-58.80) | | 0.0007 | ^c^ |
| Android body fat | 13.45 ± 3.47 | (11.00-15.90) | 14.73 ± 4.02 | (8.90-19.50) | 44.79 ± 11.39 | (29.90-55.90) | | < 0.0001 | ^b, c^ |
| Gynoid body lean | 56.25 ± 0.07 | (56.20-56.30) | 67.52 ± 4.70 | (60.90-74.91) | 47.10 ± 6.38 | (40.50-58.70) | | ns |  |
| Android body lean | 86.6 ± 3.40 | (84.20-89.00) | 85.27 ± 3.97 | (80.52-91.07) | 55.21 ± 11.39 | (44.10-69.20) | | ns |  |
|  |  |  |  |  |  |  | |  |  |
