# Supplementary material for: Early postnatal soluble FGFR3 therapy prevents the atypical development of obesity in achondroplasia
Source: PLoS One. 2018 Apr 13;13(4):e0195876. doi: 10.1371/journal.pone.0195876 (PMC5898762; doi:10.1371/journal.pone.0195876)
Supplement: S3 Table — No statistical differences were observed between the groups. (DOCX) [file pone.0195876.s006.docx]

**S3 Table. Blood parameters in the different age groups. No statistical differences were observed between the groups.**

| **Age (yrs)** | **TGO (UI/l)** | **TGP (UI/l)** | **gGT (UI/l)** | **calcium (mmol/l)** | **sodium (mmol/l)** | **potasium (mmol/l)** |
| --- | --- | --- | --- | --- | --- | --- |
| **[0-3]** | 40.33 ± 11.47 | 16.60 ± 5.68 | 9.70 ± 2.91 | 2.51 ± 0.08 | 138.40 ± 1.65 | 4.38 ± 0.23 |
| **[3-8]** | 30.92 ± 3.45 | 18.73 ± 3.88 | 13.53 ± 3.70 | 2.38 ± 0.06 | 137.69 ± 1.40 | 3.91 ± 0.23 |
| **[9-18]** | 27.58 ± 9.91 | 15.23 ± 5.43 | 12.73 ± 2.83 | 2.37 ± 0.12 | 138.08 ± 1.89 | 4.03 ± 0.47 |
|  |  |  |  |  |  |  |
|  | **bicarbonate (mmol/l)** | **phosphate (mmol/l)** | **chloride (mmol/l)** | **alcaline phosphatase (UI/l)** | **25OH vitamin D (ng/ml)** |  |
| **[0-3]** | 20.70 ± 3.68 | 1.59 ± 0.16 | 102.50 ± 2.01 | 196.10 ± 53.81 | 55.50 ± 13.66 |  |
| **[3-8]** | 22.75 ± 3.24 | 1.44 ± 0.13 | 101.50 ± 2.58 | 244.74 ± 119.7 | 38.72 ± 17.03 |  |
| **[9-18]** | 21.92 ± 1.55 | 1.17 ± 0.14 | 103.38 ± 2.72 | 258.00 ± 183.22 | 32.00 ± 4.08 |  |
