# Supplementary material for: Early postnatal soluble FGFR3 therapy prevents the atypical development of obesity in achondroplasia
Source: PLoS One. 2018 Apr 13;13(4):e0195876. doi: 10.1371/journal.pone.0195876 (PMC5898762; doi:10.1371/journal.pone.0195876)
Supplement: S4 Table — (DOCX) [file pone.0195876.s007.docx]

**S4 Table. Expression of FGFRs 1-4 in subcutaneous whit adipose tissue (scWAT), brown adipose tissue (BAT), pancreas and liver in 3 days old *Fgfr3^ach/+^* pups. Fold increase vs WT mice. 0, no statistical difference; +, 2-10 fold increase; ++, >10 fold increase; --, >10 fold decrease.**

|  | scWAT | BAT | Pancreas | Liver |
| --- | --- | --- | --- | --- |
| FGFR1 | 0 | _ _ | + | 0 |
| FGFR2 | 0 | + | ++ | + |
| FGFR3 | ++ | ++ | + | ++ |
| FGFR4 | 0 | 0 | 0 | 0 |
