# Supplementary material for: Early postnatal soluble FGFR3 therapy prevents the atypical development of obesity in achondroplasia
Source: PLoS One. 2018 Apr 13;13(4):e0195876. doi: 10.1371/journal.pone.0195876 (PMC5898762; doi:10.1371/journal.pone.0195876)
Supplement: S5 Table — (DOCX) [file pone.0195876.s008.docx]

**S5 Table.** **List of genes studied using custom RT2 Profiler PCR Array in mesenchymal stem cells.**

| **Gene Symbol** | **Official Full Name** | **Refseq #** |
| --- | --- | --- |
| Adipoq | Adiponectin, C1Q and collagen domain containing | NM_009605 |
| Cebpa | CCAAT/enhancer binding protein (C/EBP), alpha | NM_007678 |
| Cebpb | CCAAT/enhancer binding protein (C/EBP), beta | NM_009883 |
| Cebpd | CCAAT/enhancer binding protein (C/EBP), delta | NM_007679 |
| Dlk1 | Delta-like 1 homolog (Drosophila) | NM_010052 |
| Fabp4 | Fatty acid binding protein 4, adipocyte | NM_024406 |
| Fasn | Fatty acid synthase | NM_007988 |
| Gata3 | GATA binding protein 3 | NM_008091 |
| Hprt | Hypoxanthine guanine phosphoribosyl transferase | NM_013556 |
| Lep | Leptin | NM_008493 |
| Lipe | Lipase, hormone sensitive | NM_010719 |
| Lpl | Lipoprotein lipase | NM_008509 |
| Pparg | Peroxisome proliferator activated receptor gamma | NM_011146 |
| Ppargc1a | Peroxisome proliferative activated receptor, gamma, coactivator 1 alpha | NM_008904 |
| Rpl13a | Ribosomal protein L13a | NM_009438 |
| Rpl6 | Ribosomal protein L6 | NM_011290 |
| Scd1 | Stearoyl-Coenzyme A desaturase 1 | NM_009127 |
| Slc2a4 | Solute carrier family 2 (facilitated glucose transporter), member 4 | NM_009204 |
| Srebf1 | Sterol regulatory element binding transcription factor 1 | NM_011480 |
| Ucp1 | Uncoupling protein 1 (mitochondrial, proton carrier) | NM_009463 |
| Wnt1 | Wingless-related MMTV integration site 1 | NM_021279 |
